# Enhanced detection of cell-free DNA (cfDNA) enables its use as a reliable biomarker for diagnosis and prognosis of gastric cancer

**DOI:** 10.1371/journal.pone.0242145

**Published:** 2020-12-02

**Authors:** Jiyoon Bu, Tae Hee Lee, Woo-jin Jeong, Michael J. Poellmann, Kara Mudd, Hyuk Soo Eun, Elizabeth W. Liu, Seungpyo Hong, Sung Hee Hyun

**Affiliations:** 1 Pharmaceutical Sciences Division, School of Pharmacy, University of Wisconsin-Madison, Madison, WI, United States of America; 2 Department of Senior Healthcare, BK21 plus program, Graduated School, Eulji University, Daejeon, Republic of Korea; 3 Research Institute for Future Medical Science, Chungnam National University Sejong Hospital (CNUSH), Sejong, Republic of Korea; 4 Department of Biological Engineering, Inha University, Incheon, Republic of Korea; 5 Divison of Gastroenterology and Hepatology, Department of Internal Medicine, Chungnam National University Hospital, Daejeon, Republic of Korea; 6 Department of Biomedical Engineering, College of Engineering, University of Wisconsin-Madison, Madison, WI, United States of America; 7 Yonsei Frontier Lab and Department of Pharmacy, Yonsei University, Seoul, Republic of Korea; The Ohio State University, UNITED STATES

## Abstract

Although circulating cell-free DNA (cfDNA) is a promising biomarker for the diagnosis and prognosis of various tumors, clinical correlation of cfDNA with gastric cancer has not been fully understood. To address this, we developed a highly sensitive cfDNA capture system by integrating polydopamine (PDA) and silica. PDA-silica hybrids incorporated different molecular interactions to a single system, enhancing cfDNA capture by 1.34-fold compared to the conventional silica-based approach (p = 0.001), which was confirmed using cell culture supernatants. A clinical study using human plasma samples revealed that the diagnostic accuracy of the new system to be superior than the commercially available cfDNA kit, as well as other serum antigen tests. Among the cancer patients, plasma cfDNA levels exhibited a good correlation with the size of a tumor. cfDNA was also predicative of distant metastasis, as the median cfDNA levels of metastatic cancer patients were ~60-fold higher than those without metastasis (p = 0.008). Furthermore, high concordance between tissue biopsy and cfDNA genomic analysis was found, as HER2 expression in cfDNA demonstrated an area under ROC curve (AUC) of 0.976 (p <0.001) for detecting patients with HER2-positive tumors. The new system also revealed high prognostic capability of cfDNA, as the concentration of cfDNA was highly associated with the survival outcomes. Our novel technology demonstrates the potential to achieve efficient detection of cfDNA that may serve as a reliable biomarker for gastric tumor.

## Introduction

Freely circulating DNA in bloodstream has attracted a great deal of attention for its translational potential, given its role as a biomarker containing genetic information [1]. Growing clinical evidence indicates the potential of circulating cell-free DNA (cfDNA) as a reliable cancer biomarker, as demonstrated by a myriad of technologies that have been reported [2,3]. Increased cfDNA levels in blood serve as a strong indicator of malignancy, which is predicative of poor clinical outcome. The serum cfDNA levels typically remain below 200 ng/mL in healthy individuals, whereas they are significantly increased among cancer patients [4]. cfDNA monitoring has also revealed the high prognostic value of the biomarker in various tumor types, showing strong correlation with survival outcomes and patient responses to therapies [5,6]. Moreover, the genetic mutations and epigenetic alterations in cfDNA have been found to be related to the tissue of its origin [7]. It is thus obvious that sensitive detection of cfDNA and subsequent molecular analysis would enable effective therapeutic management of cancer patients in a personalized manner.

Numerous strategies have been developed to extract cfDNA from human peripheral blood [8]. DNA separation based on silica adsorption is one of the most commonly utilized strategies in various cfDNA-based liquid biopsy systems [9]. The exact mechanism behind the silica-DNA interaction is not clearly defined; however, it is known that the interaction is contributed by salt bridge formation between the negatively charged phosphate DNA backbone and the surface silanol groups, as well as hydrophobic attachment of nucleobases to non-polar region of a silica surface [10,11]. Alternatively, polydopamine (PDA), a mussel-inspired adhesive material, is being utilized as a novel DNA adsorbent. Meng et al. demonstrated that PDA nanoparticles can selectively adsorb and separate DNA from other biological components [12]. PDA catechol groups effectively adsorb DNA bases through metal coordination under the presence of polyvalent metal ions, particularly Ca^2+^ [12]. Additional π- π stacking and hydrogen bonding further enhance DNA adsorption on PDA [13]. Interestingly, every component of oligonucleotides, ranging from aromatic rings and polar functional groups of nucleobases to phosphate backbones, has potential to bind with either PDA or silica. Therefore, we expected that hybridization of silica and PDA would synergistically enhance DNA adsorption due to the two following reasons: (1) PDA would help more silica to be functionalized on a DNA capture surface, due to its strong adhesion with silica [14]; and (2) Cooperation of different molecular interactions to a single system (i.e. combined interaction of PDA-nucleobases and silica-phosphate backones), will allow for strong multivalent interaction with target molecules [15–19].

In this study, we engineered PDA-silica hybrids on biocompatible alginate beads through a layer-by-layer coating of the bead surface with PDA and silica. The bead surfaces were analyzed using X-ray photoelectron spectroscopy (XPS) and scanning electron microscopy with energy dispersive spectroscopy (SEM/EDS), in order to confirm that the strong adhesive properties of PDA allowed more silica to be functionalized onto the bead surfaces. The DNA adsorption behaviors of the PDA-silica hybrids were assessed using cell culture supernatants obtained from a gastric cancer cell line, AGS cells, which was done by comparing the amount of cfDNA adsorbed on PDA-silica hybrids with that on the control silica surfaces. We then conducted a prospective study to examine the clinical utility of cfDNA in a cohort of patients with gastric tumor. The plasma cfDNA levels in patients with malignant tumor were compared to those in healthy donors and benign tumor patients, to validate the diagnostic capability of cfDNA. Furthermore, using our newly developed system, the predictive value of cfDNA in tumor progression was also tested by correlating the amount of cfDNA with the size of the primary tumor and the metastatic potentials, while the prognostic value was investigated by correlating the cfDNA results with survival outcomes. These were then compared with the results obtained from commercially available cfDNA isolation kits, as well as other well-established serum biomarkers, including cfDNA, cfHER2 DNA, lactate dehydrogenase (LDH), c-reactive protein (CRP), carcinoembryonic antigen (CEA), and cancer antigen 19–9 (CA 19–9). We also examined human epidermal growth factor receptor 2 (HER2) gene expression in cfDNA and the results were compared with HER2 immunohistochemistry (IHC) status in biopsy, in order to verify whether the cfDNA separated using the PDA-silica hybrids carry the same molecular characteristic to that of the primary tumor. Here, by using our newly-developed cfDNA detection system, we present our results that indicate the promising potential of cfDNA as a reliable biomarker for gastric cancer.

## Materials and methods

### Materials

Sodium alginate, calcium chloride (CaCl2), silica solution (LUDOX® AM colloidal silica, 30 wt. % in H_2_O), 2-(3,4-Dihydroxyphenyl)ethylamine hydrochloride (dopamine hydrochloride), 1-(3-Dimethylaminopropyl)-3-EthylcarbodiimideHydrochloride (EDC), and N-Hydroxy-Succinimide (NHS) werei all obtained from Sigma–Aldrich (St. Louis, MO). Cell culture media RPMI-1640 medium, fetal bovine serum (FBS), and penicillin-streptomycin were purchased from Invitrogen Corporation (Carlsbad, CA). Tris(hydroxymethyl)aminomethane hydrochloride (Tris-HCl) was obtained from Millipore (Billerica, MA). Proteinase K, 95% ethyl alcohol, AW1 wash buffer, nuclease-free water, and SYBR Green Master Mix (2× Rotor Gene SYBR Green PCR Master Mix) were purchased from Qiagen Inc. (Valencia, CA). All other chemicals used in this study were obtained from Sigma-Aldrich and used without further purification unless otherwise noted.

### Synthesis of alginate beads

To prepare alginate beads, 10 mL of sodium alginate-containing solution (5% w/v in deionized water) was added into 200 mL of water containing 100 mM calcium chloride dropwise using 200 μL pipette and stirred gently at room temperature for 1 h, according to a protocol published previously [20]. The prepared beads were washed with deionized water (DW) three times (1 min for each wash) and stored in DW at 4 ˚C.

### Surface engineering of alginate beads with PDA-silica hybrids

The alginate beads were modified with dopamine hydrochloride using the EDC/NHS chemistry as described previously [16,17]. Briefly, the carboxylic groups on the alginate beads (~1 mL per bead) were activated with 5 mM of EDC/NHS at room temperature for 1 h. Dopamine hydrochloride (5 mM) was added into the bead-containing solution dropwise and stirred at room temperature for 12 h. Acidity of the dopamine hydrochloride solution was maintained at pH of ~6.5 using Tris-HCl buffer. The dopamine-functionalized beads were then washed with DW for three times (1 min for each) and mixed with 1 mL silica solution for 1 h at room temperature. The resulting PDA-silica-coated alginate beads were immediately used for cfDNA capture.

### Surface characterization using XPS

The chemical composition of the beads was identified by XPS using MultiLab 2000 (Thermo VG Scientific, West Sussex, UK) equipped with a monochromatic AlKα excitation source (hυ = 1486.6 eV). Survey spectra were collected over a range of 0–1,200 eV with pass energy of 100.0 eV and a step of 1.0 eV. The take-off angle of the photoelectron was set at 35°. High-resolution spectra of C_1s_, O_1s_ and Si_2p_ were also collected under adjusted condition (30.0 eV and 0.1 eV) and the binding energy (BE) scale was calibrated by comparing with the neutral adventitious C_1s_ peak at 284.6 eV. Each measurement was repeated for five times.

### Surface characterization using SEM/EDS

The morphology of the beads was examined by SEM/EDS using SU8200 (Hitachi, Tokyo, Japan), as described previously [21]. Surface morphology was obtained via SEM mode at an acceleration voltage of 2 kV and with a working distance of 4.7 mm. All images were magnified by a factor of 1 K. The prepared bead samples were attached to a double-sided adhesive tape mounted on the SEM stub and coated with osmium (3.0 nm in thickness) in order to avoid degradation or charging effects. In addition, elemental composition and distribution were analyzed by an EDS mode using an Octane Elite Super EDS System (AMETEK, Inc., Paoli, PA) that incorporates a silicon nitride (Si_3_N_4_) window. The EDS mapping was achieved at 10 kV with a resolution of 124.5 eV for 30 s. The take-off angle of the photoelectron was set at 29.1°. Each measurement was repeated three times for the selected area, and the results were analyzed using a TEAM™ EDS Analysis System (AMETEK, Inc.).

### Preparation of cell culture supernatant

A human gastric cancer cell line, AGS, was obtained from the Biobank of Chungnam National University Hospital, which was purchased from Korea Cell Line Bank (Seoul, Republic of Korea) on July 2018. Cells were frozen in aliquots at passage ≤ three and stored in liquid nitrogen. Experiments were conducted after three passages post thaw (passage number ≤ 6), within a year from the purchase. Cells were grown continuously as a monolayer under a humidified condition at 37°C, 5% CO_2_ in RPMI-1640 medium supplemented with 10% (v/v) FBS and 1% (v/v) penicillin/streptomycin. Cell culture media were replaced when the cells reached at 70% confluency. For cfDNA analysis, the cell culture media were transferred to conical tubes after 24 h incubation, followed by a relatively gentle centrifugation step at 1,500 g for 3 min to remove cells and large debris in the supernatants. The supernatants were collected and immediately used for the cfDNA analysis. All experiments were performed with mycoplasma-free cells, which was confirmed by DAPI staining.

### Plasma cfDNA analysis

This study was approved by an institutional review board (IRB) of Eulji University, Daejeon, Korea (EU18-68). The blood samples and clinical data were provided from the National University Hospital Biobanks of Chungbuk and Chonbuk, members of the Korea Biobank Network. Human blood samples were obtained by venipuncture and collected in 3 mL BD Vacutainer® K2EDTA anticoagulant tubes (Becton Dickinson, Franklin Lakes, NJ). The cellular components were depleted from the whole blood within 5 hours of blood draw, which was done by two-step centrifugation: 1,700 g for 10 min, followed by 300g for 15 min [22]. Plasma layers were gently obtained and apportioned into 200 μL aliquots. The samples were stored at -80˚C until use.

Each aliquot was treated with proteinase K at a 10:1 (v/v) ratio and mixed with 200 μL lysis buffer. The samples were incubated at 37 ˚C for 10 min, followed by the reaction with 200 μL of 95% ethanol. A single PDA-silica-coated bead was then added into the sample (total 620 μL) and mixed with 5 μL calcium chloride solution, followed by incubation under gentle agitation for 10 min, allowing DNA fragments to be captured on PDA-silica surface. The beads were washed with 350 μL AW1 wash buffer and stored in 50 μL RNase/DNase free water. Plasma cfDNA concentrations were assessed using Bioanalyzer 2100 (Agilent Technologies, Santa Clara, CA). All processes were performed at room temperature. Lamda DNA (Thermo Scientific) was used as standard. For comparison, QIAamp DNA mini-kit was also employed for DNA extraction and compared with our system, following the manufacturers’ instruction.

### Real-time quantitative polymerase chain reaction (qPCR)

A Rotor Gene 6200 real time cycler and its software (Corbett Research, Sydney, Australia) were utilized for the gene amplification and data collection, respectively. Real-time qPCR was performed in triplicate using SYBR Green Master Mix. All reactions were performed in a final volume of 25 μL. The PCR cycling condition involves 95˚C for 5 min with 40 cycles, 95˚C for 5 s, and 60˚C for 10 s. cfHER2 DNA expression was obtained by comparing the *c-erb*B-2 (HER2) gene expression with the reference gene, ribonuclease P/MRP subunit P30 (*RPP30*) (2^-ΔCt^). Ct values above 40 were excluded for the data analysis. The primer sequences used are as following: HER2 forward primer: 5’-CCTCTGACGTCCATCATCTC-3’, HER2 reverse primer: 5’-ATCTTCTGCTGCCGTCGCTT-3’, RPP30 forward primer: 5’-GATTTGGAC CTGCGAGCG-3’, and RPP30 reverse primer: 5’-GCGGCTGTCTCCACAAGT-3’. The cut-off value of HER2/RPP30 for the survival analysis was determined as 2.5, according to a previous report [23].

### Statistics

The diagnostic/predictive values of the peripheral blood biomarkers, including cfDNA (obtained from PDA-SiO2 beads and existing kits), cfHER2 DNA, LDH, CRP, CEA, and CA 19–9, were examined by comparing the amount of each biomarker between different subgroups; i.e. patients vs. healthy donors or depending on TNM stages. The analysis was performed using Student’s *t* test or Mann-Whitney *U* test, depending on the normality of the data. Clinical indications of these biomarkers were further analyzed by constructing a receiver operating characteristic (ROC) curve. The prognostic value of cfDNA was validated using the Kaplan-Meier plots and the Cox Regression model for both overall survival (OS) and disease-free survival (DFS). A value of p <0.05 was regarded as statistically significant. All statistical analyses were conducted using SPSS Statistics 24 (SPSS Inc, Chicago, IL).

## Results and discussion

### cfDNA isolation using PDA-silica beads

**Fig 1:**
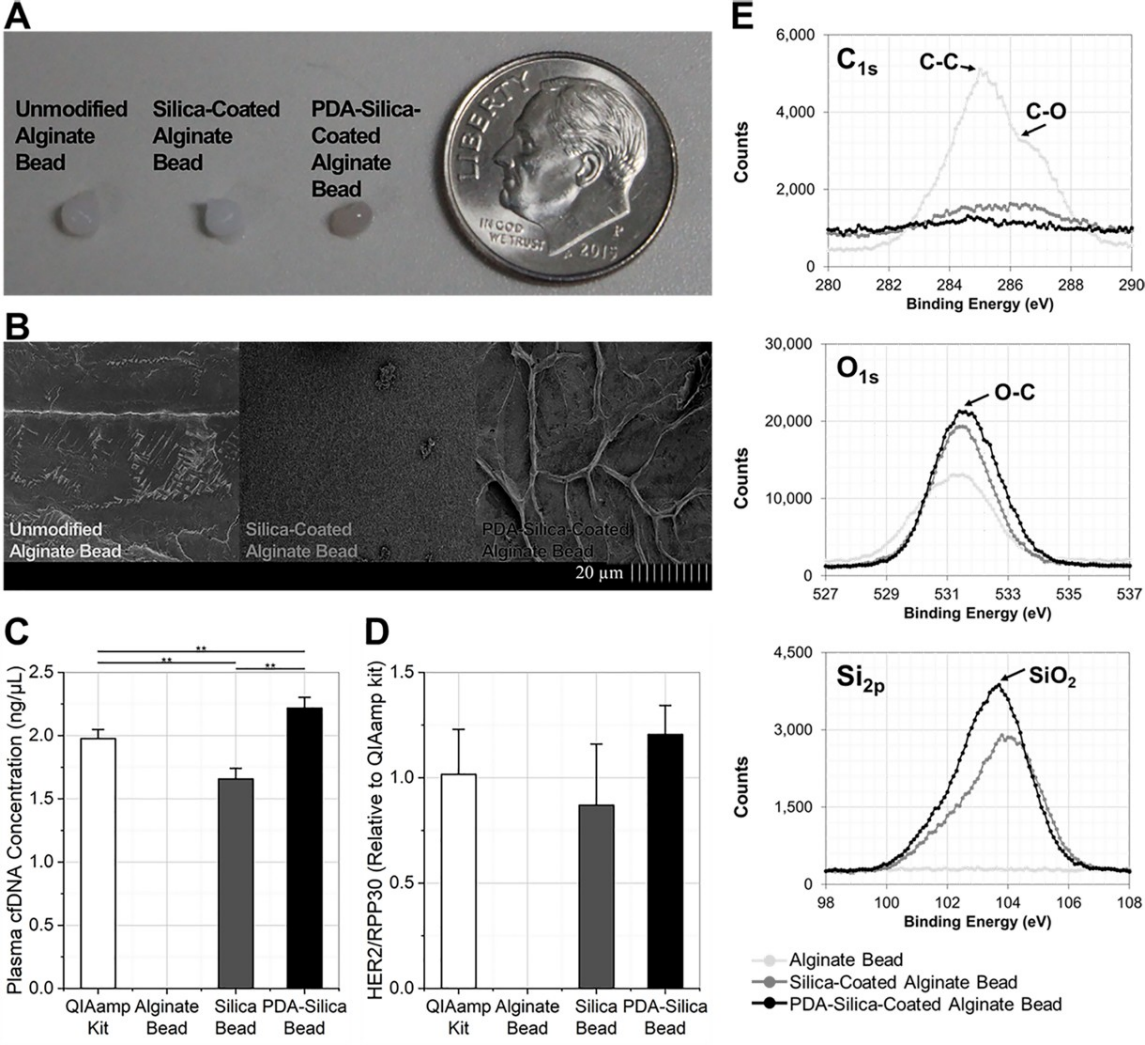
PDA-silica hybrids for enhanced cfDNA adsorption. (a) PDA-silica hybrids functionalized layer-by-layer on an alginate bead, having a diameter of ~4 mm. (b) Morphological modification confirmed by scanning electron microscopy (SEM) after coating PDA-silica hybrids on the bead surface. (c) cfDNA adsorption from each assay compared using cell culture supernatants obtained from a gastric cancer cell line, AGS. PDA-silica hybrids showed enhanced cfDNA adsorption by 1.12-fold (p = 0.019) and 1.34-fold (p = 0.001) compared to the QIAamp DNA mini kit and silica-coated alginate beads, respectively. (d) The relative amount of cfHER2 DNA quantified from each assay. Both the DNA fragments obtained by PDA-silica hybrids and silica showed significant cfHER2 DNA expression. (e) X-ray photoelectron spectroscopy (XPS) analysis demonstrating the presence of PDA and silica on the surface of PDA-silica alginate beads. PDA-silica beads exhibited the highest SiO_2_ (~103.5 eV) peak on Si_2p_ spectra, indicating that PDA incorporation displays a benefit for enhancing silica coating on alginate beads.

Enhanced cfDNA adsorption to the PDA-silica beads was confirmed using cell culture supernatants of AGS cells, a gastric cancer cell line (Fig 1A and 1B). cfDNA levels were assessed using three different types of beads (unmodified, silica-coated, and PDA-silica-coated alginate beads) and a commercial cfDNA extraction kit, QIAamp DNA mini kit, that purifies nucleic acids using silica-membranes. Fig 1C shows the amount of cfDNA extracted using each assay. cfDNA was not detected on the unmodified alginate beads, indicating that alginate itself does not interact with cfDNA at a detectable level. Notably, the PDA-silica beads exhibited the highest cfDNA levels, improving the cfDNA adsorption by 1.12-fold (p = 0.019) and 1.34-fold (p = 0.001) compared to the QIAamp DNA mini-kit and the silica-coated beads, respectively. The relative amount of cfHER2 DNA was analyzed and compared to RPP30 reference gene expression, in order to confirm that cfDNA expresses the tumor-specific genes that are derived from its parental cells (Fig 1D). There was no significant difference in relative HER2 expression among the nucleic acids detected using the three methods employed. This result reveals that the DNA fragments adsorbed on the PDA-silica hybrids are not different from those captured on the conventional silica-based DNA extraction method, and that the detected DNA reflect the biological characteristics of their parental cells [24]. Collectively, the PDA contributes to cfDNA capture, exhibiting higher sensitivity in detecting cfDNA than the conventional silica-based cfDNA adsorption.

We have hypothesized that the strong adhesion properties of PDA would enrich the silica on the alginate surface, thereby increasing the cfDNA adsorption capacity of the silica-based system [25], in addition to its direct interaction with DNA bases through metal coordination [12]. We assessed this hypothesis by comparing chemical compositions of the three different beads using XPS and SEM/EDS analysis (Fig 1E and S1 Fig). Fig 1E shows the high-resolution spectra of C_1s_, O_1s_ and Si_2p_ after different surface modification processes. The C_1s_ spectrum of the unmodified alginate beads showed typical C-O (~286.0 eV) and C-C (~284.8 eV) peaks, which are characteristic peaks corresponding to the crosslinked alginate beads. Upon incorporation of DNA adsorbents onto the surface of the beads, these peaks disappeared, confirming the surface modification. It is noteworthy that the PDA-silica beads displayed a weaker signal of C-C bond in the C_1s_ spectrum compared to the silica-beads, inferring that the PDA-silica hybrids covered the alginate bead surface more effectively than silica alone. Our results obtained from the O_1s_ and Si_2p_ spectra further support this hypothesis, as both O-C (~531.5 eV) and SiO_2_ (~103.5 eV) peaks from the PDA-silica beads were higher than those from the bead with silica only. These results further confirm that PDA plays a key role in incorporating additional molecular interactions, increasing the amount of silica present on the beads, and maximizing cfDNA adsorption.

### Diagnostic capability of cfDNA adsorbed on PDA-silica beads

A total of 85 patients with gastric tumor, 61 with malignant tumor and 24 with benign tumor, and 17 healthy individuals were enrolled in this pilot study. The majority (49 out of 61) of the patients with malignant tumors were in advanced stages (T≥3). All patients involved in our study underwent surgical resectioning after the blood draw. Clinical information including histopathological stage, histology type, cell differentiation, cancer-associated marker expression, and serum antigen levels were determined based on the physical exam, computed tomography (CT) scan, biopsy, IHC, and/or blood tests (S1 Table). Preoperative plasma cfDNA levels were quantitatively measured using Bioanalyzer.

**Fig 2:**
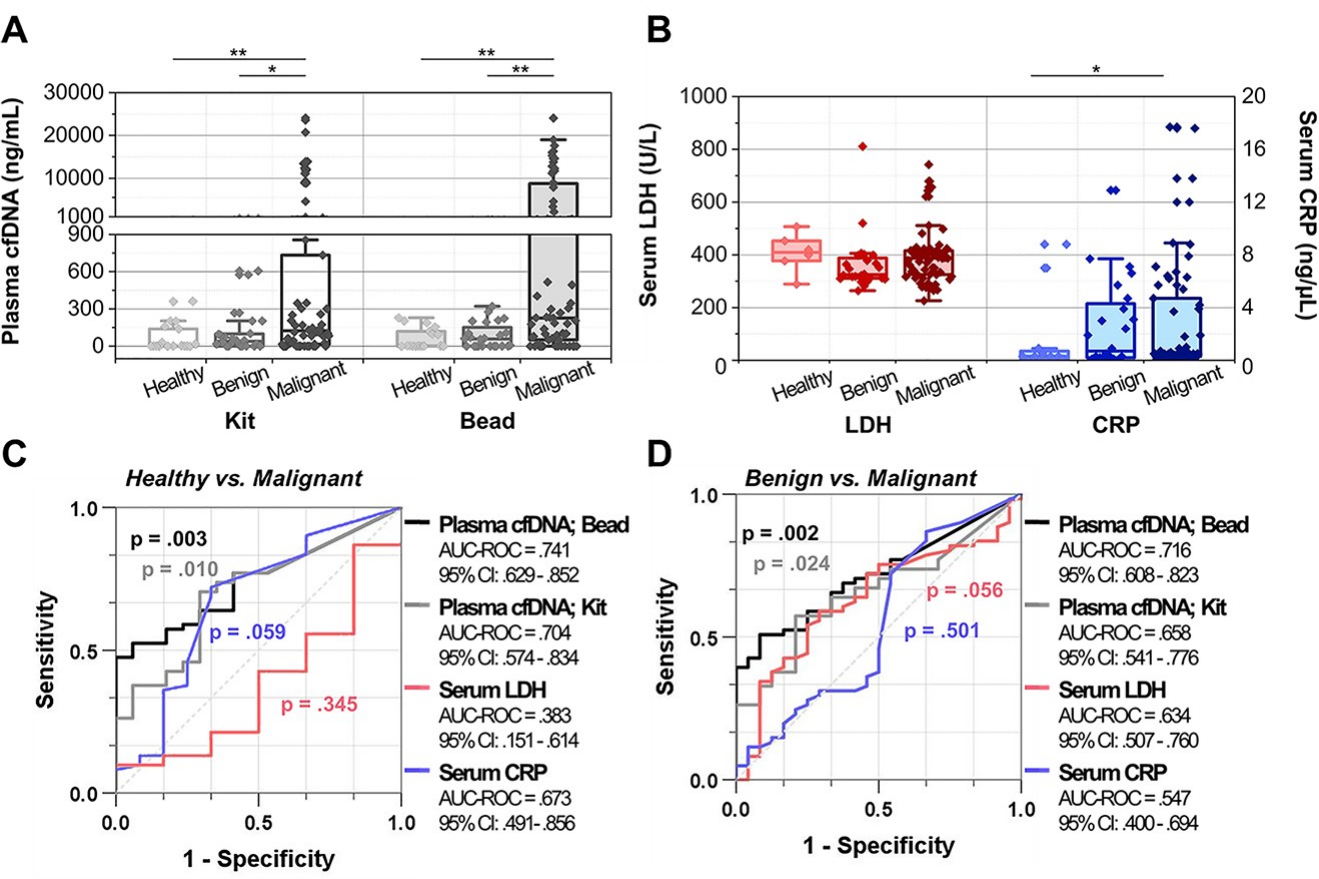
Plasma cfDNA and serum antigen levels quantified from a total of 85 patients with gastric tumor, 61 with malignant tumor and 24 with benign tumor, and 17 healthy individuals. (a) The amount of plasma cfDNA levels measured from each cohort using the new bead-based cfDNA separation system and commercially available QIAamp DNA mini kit. (b) The amount of serum LDH and CRP levels measured from each cohort. (c, d) The diagnostic capability of the new cfDNA separation platform compared with commercially available QIAamp DNA mini kit and other serum antigen blood tests. The new bead-based system demonstrated the highest performance in distinguishing the patients with malignant tumor from both (c) healthy donors and (d) patients with benign tumor.

We tested our PDA-silica-based cfDNA separation system using human plasma samples and compared the concentrations of detected cfDNA with those obtained using QIAamp DNA mini-kit (Fig 2A). The representative electropherograms and gel-like images of cfDNA obtained using our newly developed system and QIAamp DNA mini-kit are presented in S2 Fig. In case of PDA-silica-coated beads, median plasma cfDNA concentration (interquartile range; IQR) of the patients with malignant gastric tumor was 225.8 (50.8–8760.8) ng/mL. This was significantly higher than those obtained from the patients with benign tumor and healthy individuals, which exhibited median plasma cfDNA concentrations of 58.3 (0.0–129.6) ng/mL (p = 0.002) and 3.3 (0.0–118.3) ng/mL (p = 0.002), respectively. QIAamp DNA mini kit also demonstrated higher cfDNA concentration for the patients with malignant tumor, as median cfDNA levels were measured to be 123.3 (15.8–733.3) ng/mL, 18.3 (0.0–100.8) ng/mL, and 0.8 (0–138.3) ng/mL for the patients with malignant tumor, patients with benign tumor, and healthy individuals, respectively. However, the differences were less pronounced compared to PDA-silica beads, showing p values of 0.022 and 0.010 for the patients with malignant tumor vs. patients with benign tumor and healthy individuals, respectively.

The diagnostic capability of cfDNA adsorbed on the PDA-silica-coated beads was further compared with that of CRP and LDH (Fig 2B)–two proteins known to be elevated in a serum of cancer patients [26,27]. Slight differences were found between serum CRP levels of healthy donors (1.57 ± 2.99 mg/L) and patients with malignant tumor (2.84 ± 4.24 mg/L) at a p value of 0.049. However, there was no significant difference in CRP level between the patients with malignant and benign tumors (patients with benign tumor: 2.60 ± 3.34 mg/L; p = 0.498). The LDH concentrations exhibited the indistinguishable clinical relevance in our cohort, as all three groups demonstrated similar LDH levels with no statistical significance.

The ROC curves were then constructed to quantitatively assess the diagnostic performance of our PDA-silica-based cfDNA separation system (Fig 2C and 2D). Our new cfDNA analysis system exhibited a significant diagnostic capability by discriminating malignant tumor patients from healthy individuals and patients with benign tumor with an area under ROC curve (AUC-ROC) of 0.741 (p = 0.003) and 0.716 (p = 0.002), respectively. These were statistically more accurate than the QIAamp mini kit, having AUC-ROC of 0.704 (p = 0.010) and 0.658 (p = 0.024), respectively. Furthermore, ROC analysis of serum LDH and CRP levels revealed that cfDNA may be a superior biomarker that can be detected at higher sensitivity/specificity compared to conventional serum antigen analysis, since these methods exhibited AUC-ROC of below 0.680 with substantially less statistical significance (p>0.056). Note that the difference in age and gender did not affect the expression of tumor biomarkers, implying that the effects of difference in age (S2 Table) and proportion of gender (S3 Table) between the cohorts are negligible.

### Predictive capability of cfDNA for estimating the size and metastatic potential of a tumor

**Fig 3:**
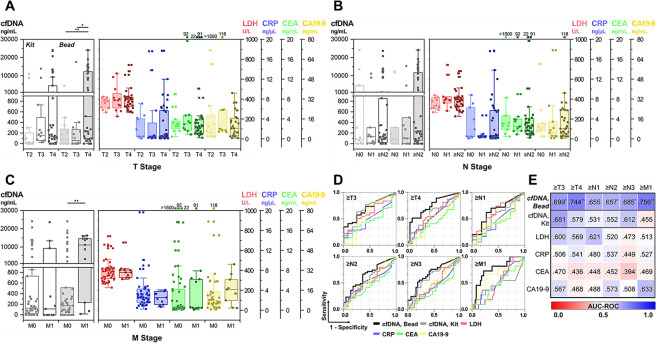
Plasma cfDNA levels depending on TNM stage. (a-c) The amount of plasma cfDNA measured using the new bead-based system and commercially available QIAamp DNA mini kit, along with serum antigen expressions depending on (a) T, (b) N, and (c) M stage. (d) The ROC curve analysis demonstrating clinical capability of the new cfDNA separation system in discriminating cancer patients depending on the TNM stage.

Patients with malignant tumors were stratified based on their tumor-node-metastasis (TNM) stages (S4 Table). Fig 3A depicts the plasma cfDNA levels of each subgroup based on the tumor size and extent (T stage). The measured amounts of DNA fragments from PDA-silica-coated beads were indicative of the advanced T stage, showing median cfDNA levels of 515.8 (98.3–12313.3) ng/mL for T4 cancer patients, which was significantly higher than those of T2 (78.3 (0–275.8) ng/mL; p = 0.007) and T3 patients (59.6 (0–247.7) ng/mL; p = 0.018). In contrast, plasma cfDNA levels measured using QIAamp DNA mini kit or other serum biomarkers, which include LDH, CRP, CEA, and CA19-9, generated poor correlation with T stage. Patients with higher T stage appeared to have, in general, greater levels of these tumor biomarkers in serum; however, the results were statistically insignificant (p >0.050). The results were examined more specifically by analyzing the correlation between the amount of plasma cfDNA and size of tumor burden. As shown in S3 Fig, the amount of DNA fragments on PDA-silica-coated beads showed a positive correlation with the size of tumor burden, with Pearson’s correlation coefficient of 0.352 (p = 0.005). In contrast, the results obtained from QIAamp DNA mini kit or other serum antigen tests demonstrated the correlation coefficients below 0.238 (p >.05), revealing that none of these tests was indicative of tumor burden.

The prognostic value of the new bead-based cfDNA separation system to detect distant and regional lymph node metastasis was also investigated (Fig 3B and 3C). A total of 11 patients (18.0%) recruited in this study developed distant metastasis; 9 of which were diagnosed with metastatic disease, and the other 2 patients subsequently developed distant metastasis after the blood draw. The plasma cfDNA levels obtained from the new system were significantly higher among patients with metastatic tumor (11178.3 (1798.3–14580.8) ng/mL), compared to those with non-metastatic tumor (180.8 (0–515.8) ng/mL). In contrast, none of the other blood tests showed any significant correlation with the development of distant metastasis. The new bead-based system, however, was less successful in detecting metastasis to the regional lymph nodes. Differences in plasma cfDNA levels were not statistically significant depending on the existence of the nodal metastasis. However, cancer patients who had tumor spreading to more number of lymph nodes (≥N2 patients; median: 233.3 ng/mL) appeared to have greater levels of plasma cfDNA than those with low-to-no lymph nodal metastasis (≤N1; median: 98.3 ng/mL), at a p value of 0.041. It should be also noted that other blood biomarkers used in this study did not have any noticeable trend depending on the number of lymph node metastasis.

The ROC analysis was performed to demonstrate the diagnostic capability of the new bead-based system for detecting the cancer patients with higher TNM stages (Fig 3D and 3E). The new cfDNA detection system highly outperformed QIAamp DNA mini kit, as well as other antigen blood tests, demonstrating the highest AUC-ROC value for differentiating patients with high TNM stages. Particularly, the new system exhibited AUC-ROC of 0.744 (p = .001), 0.657 (p = .043), and 0.756 (p = .008) for discriminating ≥T4, ≥N2, and metastatic patients, respectively, whereas the results obtained from other blood tests were statistically irrelevant with TNM stage of the patients. These imply that sensitive capture and detection of circulating DNA fragments using our platform may likely provide clinically significant information for estimating the size, aggressiveness, and metastatic potential of a tumor.

### Prognostic capability of cfDNA for estimating survival outcomes

**Fig 4:**
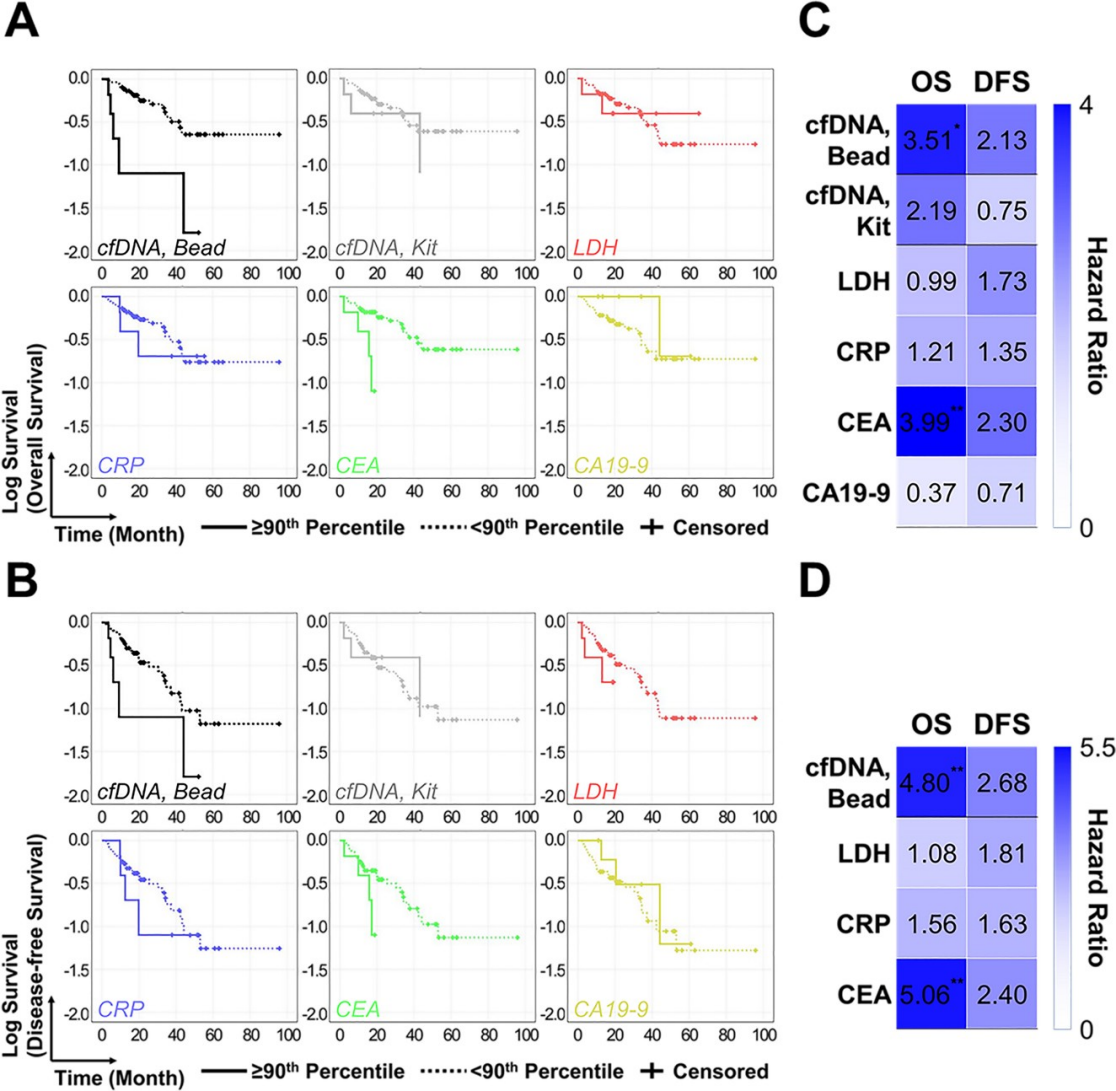
Kaplan Meier survival analysis and Cox regression analysis for demonstrating the prognostic and predictive value of the new cfDNA separation platform for patients with gastric cancer. (a, b) Kaplan Meier survival analysis of different blood tests. 90^th^ percentile was set as a cutoff for each test. (c, d) Cox regression analysis of different blood test. 90^th^ percentile was set as a cutoff for each test.

Correlation between the amount of plasma cfDNA and patients’ survival was validated using Kaplan-Meier survival analysis and Cox proportional hazard regression analysis. We initially divided the patients into two subgroups by setting the median amount of each plasma/serum biomarker as a cutoff, but none of the blood tests used in this study showed relevance with the survival outcomes. However, when increasing the cutoff values to 90^th^ percentile, cfDNA adsorbed on the PDA-silica-coated beads demonstrated a high degree of correlation with overall survival (OS). The mean OS of patients with lower cfDNA levels was 62.2 ± 6.1 months, which was significantly longer than that of the subgroups with the higher cfDNA levels (20.1 ± 8.2 months; p = .009) (Fig 4A). Similarly, disease-free survival (DFS) of the low cfDNA subgroup patients was 2.3-fold longer than that of the high cfDNA subgroup, although the results were statistically less significant (46.1 ± 5.8 months vs. 20.1 ± 8.2 months; p = 0.118) (Fig 4B). The prognostic capability of the new bead-based system was superior to that of QIAamp DNA mini kit. OS and DFS for patients with lower vs. higher cfDNA concentration were 61.9 ± 6.0 months vs. 30.6 ± 8.8 months (p = 0.144) and 45.7 ± 5.9 months vs. 30.6 ± 8.8 months (p = 0.592), respectively. For other blood antigen tests, high serum CEA levels were indicative of poor prognosis, as OS and DFS between the patients with lower vs higher cfDNA levels were determined as 63.0 ± 6.0 months vs. 22.0 ± 7.2 months (p = 0.004) and 46.8 ± 5.9 months vs. 22.0 ± 7.2 months (p = 0.082). Meanwhile, other serum antigens exhibited poor prognostic value in predicting survival outcomes.

Univariate Cox regression analysis was performed for each test as shown in Fig 4C. The amounts of cfDNA adsorbed on PDA-silica-coated beads were strongly associated with OS, demonstrating hazard ratio (HR) of 3.51 (95% CI = 1.28–9.60; p = 0.014). This outperformed QIAamp DNA mini kit which had HR of 2.19 (95% CI = 0.74–6.46; p = 0.155). For other blood antigen tests, serum CEA level exhibited HR of 3.99 (95% CI = 1.46–10.96; p = 0.007), whereas other antigen tests demonstrated weak or poor correlation with OS. In case of DFS, the new bead-based cfDNA isolation system and serum CEA measurement were the only two tests that had HR above 2, while the results were statistically less significant (p >0.05). The HR values were further adjusted by constructing the multivariate Cox regression model. Note that the cfDNA results obtained from QIAamp mini kit was excluded in multivariate Cox regression analysis, as the result of two different cfDNA tests may affect each other. Serum CA19-9 was excluded as well since the amount of CA19-9 was unmeasured from 9 patients. As shown in Fig 4D, cfDNA exhibited an effective prognostic factor for OS (HR = 4.80; 95% CI = 1.63–14.16; p = 0.004) and DFS (HR = 2.68; 95% CI = 0.98–7.34; p = 0.056). Adjusted HR of serum CEA was also similar with that of cfDNA, while other biomarkers were statistically irrelevant with patients’ survival. Collectively, these results confirmed that cfDNA captured using our PDA-silica hybrid system effectively prognoses patients with gastric tumor.

### Concordance of HER2 expression between cfDNA and tissue

**Fig 5:**
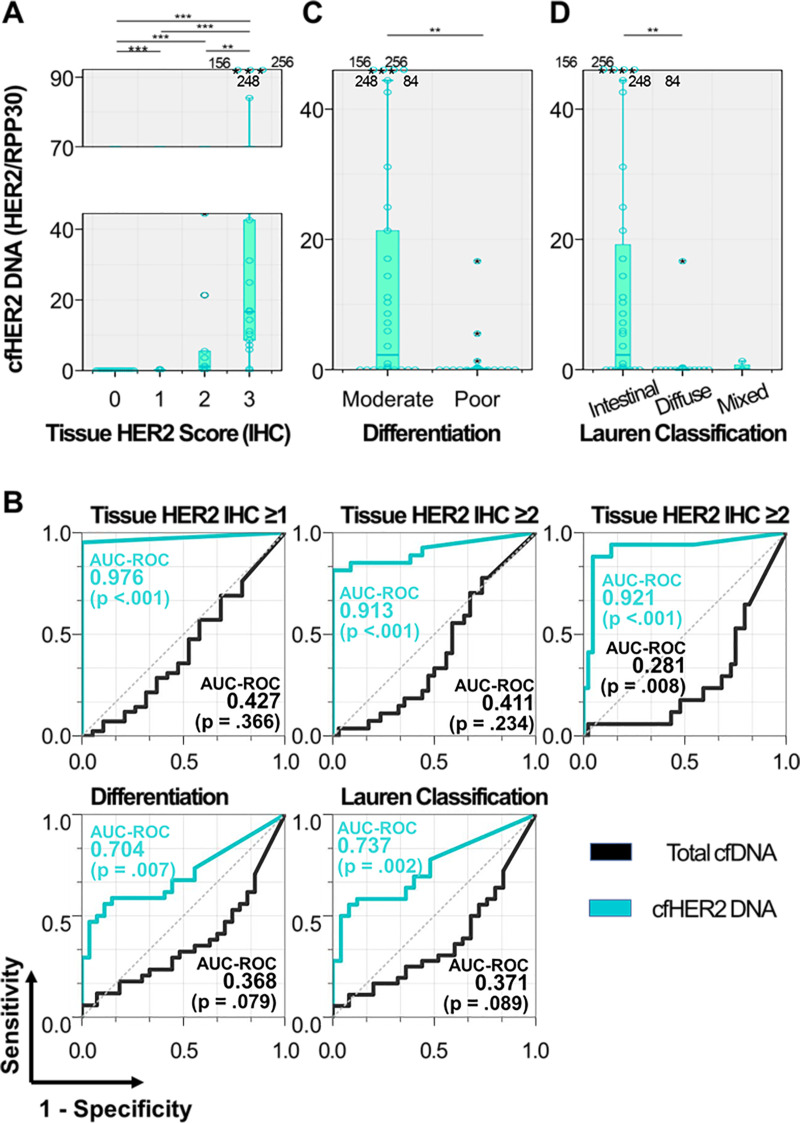
cfHER2 DNA detected from patients with malignant tumor. (a) HER2 expression in cfDNA depending on the tissue HER2 score. HER2 expression (b) ROC curve analysis demonstrating the correlation of cfHER2 DNA expression with tissue HER2 score, cell differentiation, and Lauren classification. (c, d) HER2 expression in cfDNA depending on (c) cell differentiation and (d) Lauren classification.

HER2 expression in cfDNA was investigated based on a real-time qPCR (Fig 5A). The amounts of circulating HER2-expressing gene were highly correlated with HER2 status of the primary tumor, which was defined by IHC analysis. Remarkably, HER2-expressing cfDNA (cfHER2 DNA) was not detected from all HER2-negative cancer patients (0/19), whereas it was detected in 95% (40/42) of patients having HER2-positive tumor, with a median HER2/RPP30 expression (2^-ΔCt^) of 0.70 (range: 0.00–256). Among the patients with HER2-positive tumors, cfHER2 DNA expression was closely associated with the tissue HER2 scores, exhibiting median HER2/RPP30 expression of 0.13, 1.12, and 16.65 for patients with tissue HER2 scores of 1+, 2+, and 3+, respectively. The ROC curve analysis further revealed that cfDNA strongly reflected the molecular characteristics and pathological HER2 status of primary tumors (Fig 5B). Detection of cfHER2 DNA effectively differentiated patients with HER2-presenting tumor from those with HER2-negative tumor, as shown in an AUC-ROC of 0.976 (p <0.001).

It has been well established that HER2 is more widely expressed on intestinal type gastric tumors compared to diffuse type tumors [28]. For this reason, cfHER2 DNA could serve as a good prognostic biomarker to distinguish the two groups of patients, i.e., intestinal vs. diffuse type gastric tumors [29]. We thus explored how plasma cfDNA levels and its HER2 expressions are affected by tumor differentiation (Fig 5C and 5D). Patients with well-differentiated, intestinal type gastric tumors showed higher cfHER2 DNA expressions compared to those with poorly differentiated (median: 2.27 vs. 0.08; p = 0.006), diffuse/mixed type tumors (median: 2.27 vs. 0.00; p = 0.002). cfHER2 DNA exhibited AUC-ROCs of 0.704 (p = 0.007) and 0.737 (p = 0.002) for discriminating poorly differentiated and intestinal type tumors, respectively (Fig 5B). These results indicate that our cfDNA capture platform allows reliable assessment on cancer-associated HER2, which could be potentially expanded to other oncogenes. The aspect of cfDNA captured using our system could be also expanded to other applications, such as monitoring therapeutic responses to specific treatment, thereby realizing personalized medicine.

## Conclusion

In this study, we tested the potential of cfDNA as a clinically reliable biomarker of gastric tumors. We prepared a novel platform for enhanced cfDNA adsorption by coating alginate beads with an engineered PDA-silica mixture. The comparison among various alginate bead configurations revealed that our PDA-silica-coated alginate beads achieve the highest sensitivity to cfDNA from gastric cancer cells. The DNA fragments adsorbed on the PDA-silica hybrids carried the biological characteristics of the cells they originated from, exhibiting the similar HER2 expression levels. The larger difference in cfDNA concentration between patients with malignant and benign tumors, than in CRP and LDH, clearly indicated the superior diagnostic potential of cfDNA to the conventional blood-circulating biomarkers. Moreover, a significant correlation was found between plasma cfDNA levels and tumor size/metastasis/OS, indicating its prognostic capability to distinguish cancer patients with high TNM stages from those with benign or lower TNM stages. All these results led us to conclude that cfDNA detected using our PDA-silica hybrid platform could serve as an efficient clinical indicator of gastric tumors. Our data herein provide clinical evidence that cfDNA is a promising biomarker for diagnosis and prognosis of gastric tumor, which may potentially provide actionable information to physicians.

## Supporting information

S1 Fig Chemical composition of the three different alginate bead surfaces: Unmodified alginate beads, silica-coated alginate beads, and PDA-silica-coated alginate bead. The surfaces were analyzed using SEM/EDS. Strong adhesion property of PDA enriched silica on the alginate surface.
(TIF)

S2 Fig The representative (a) electropherograms and (b) gel-like images obtained from a healthy donor and two cancer patients: cfDNA was obtained using either PDA-silica-coated alginate beads or QIAamp DNA mini-kit.
(TIF)

S3 Fig Pearson’s correlation analysis between the amounts of cancer-associated blood biomarkers and size of tumor burden. cfDNA was the only biomarker that showed statistically significant correlation with the size of tumor burden.
(TIF)

S1 Table Clinical and demographic characteristics of recruited cancer patients. (PDF)

S2 Table Pearson’s correlation analysis between the patients’ age and expression levels of different blood biomarkers. (PDF)

S3 Table Pearson’s correlation analysis between the patients’ gender and expression levels of different blood biomarkers. (PDF)

S4 Table Concentration of cancer-associated blood biomarkers depending on TNM stage. (PDF)

S1 File Clinical datasets required to replicate all findings reported in this article. (ZIP)
