# Supplementary figures and images for: Enhanced detection of cell-free DNA (cfDNA) enables its use as a reliable biomarker for diagnosis and prognosis of gastric cancer
Source: PLoS One. 2020 Dec 2;15(12):e0242145. doi: 10.1371/journal.pone.0242145 (PMC7710035; doi:10.1371/journal.pone.0242145)

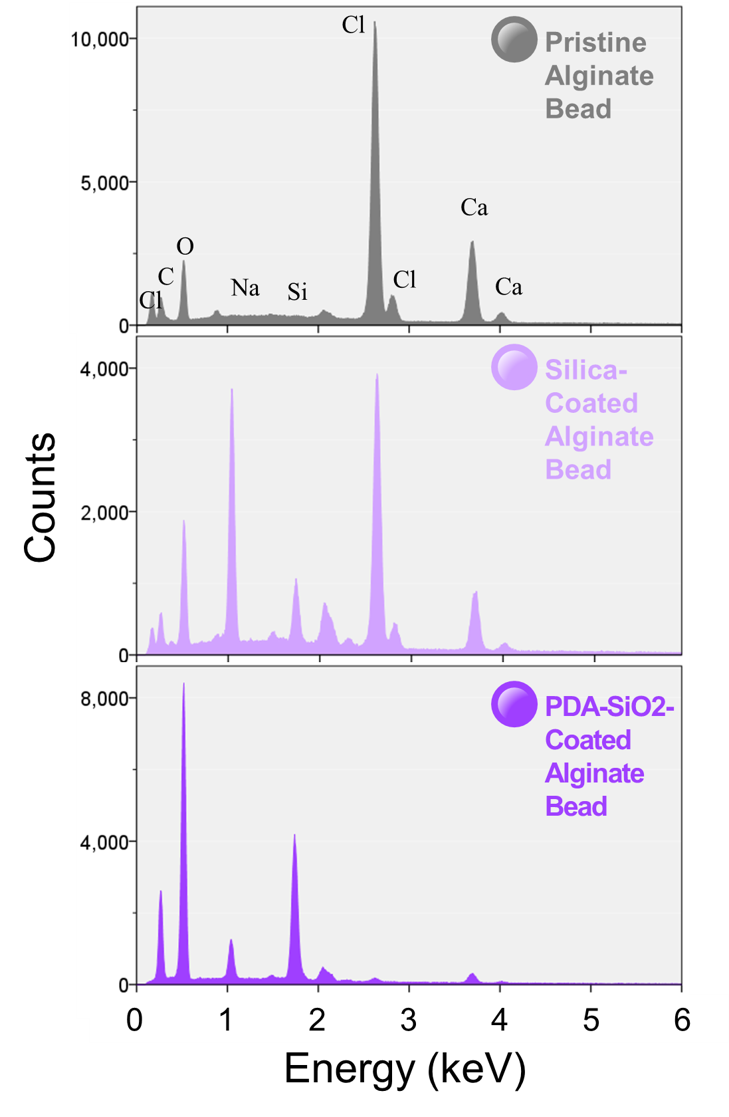

Supplement: S1 Fig — The surfaces were analyzed using SEM/EDS. Strong adhesion property of PDA enriched silica on the alginate surface. (TIF) [file pone.0242145.s001.tif]

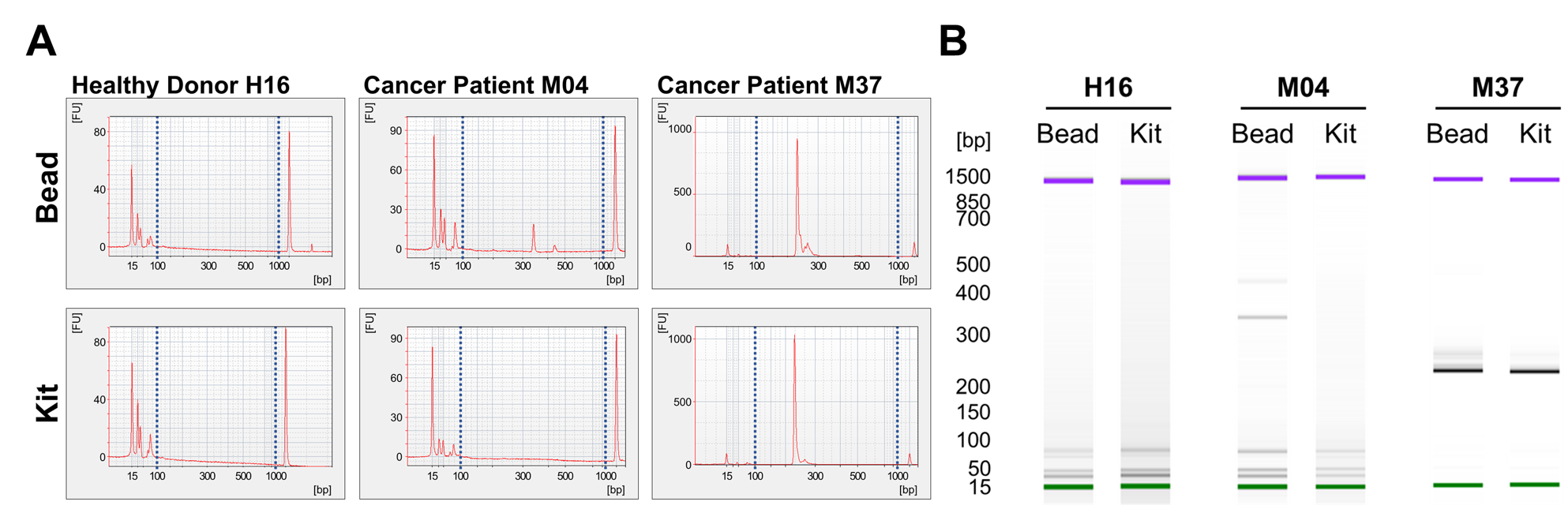

Supplement: S2 Fig — The representative (a) electropherograms and (b) gel-like images obtained from a healthy donor and two cancer patients: cfDNA was obtained using either PDA-silica-coated alginate beads or QIAamp DNA mini-kit. (TIF) [file pone.0242145.s002.tif]

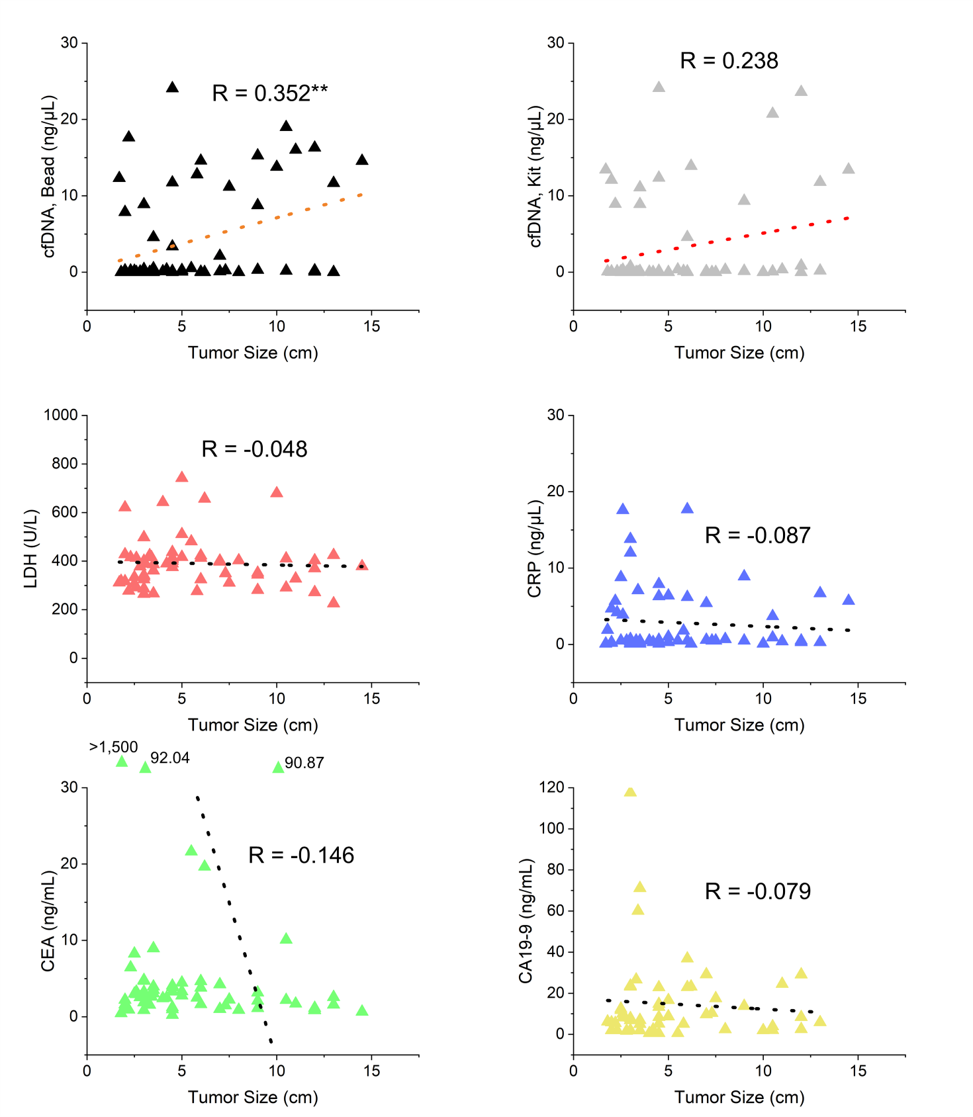

Supplement: S3 Fig — cfDNA was the only biomarker that showed statistically significant correlation with the size of tumor burden. (TIF) [file pone.0242145.s003.tif]
