# Supplementary material for: Enhanced detection of cell-free DNA (cfDNA) enables its use as a reliable biomarker for diagnosis and prognosis of gastric cancer
Source: PLoS One. 2020 Dec 2;15(12):e0242145. doi: 10.1371/journal.pone.0242145 (PMC7710035; doi:10.1371/journal.pone.0242145)
Supplement: S1 Table — (PDF) [file pone.0242145.s004.pdf]

	Patients with Malignant Tumor (n = 61)	
	No.	%
Age at blood draw		
Median	66	
Range	40-83	
Gender		
Female	20	32.8
Male	41	67.2
T Stage		
T2	12	19.7
T3	12	19.7
T4	37	60.7
N Stage		
N0	11	18.0
N1	11	18.0
N2	10	16.4
N3	29	47.5
M Stage		
M0	50	82.0
M1	11	18.0
Differentiation		
Poor	27	44.3
Moderate (+ Intermediate)	34	55.7
Histological Subtype		
Papillary Adenocarcinoma	4	6.6
Poorly Cohesive Adenocarcinoma	3	4.9
Poorly Cohesive Carcinoma	17	27.9
Tubular Adenocarcinoma	37	60.7
Lauren's Classification		
Diffuse	22	36.1
Mixed	3	4.9
Intestinal	36	59
Tissue HER2 Score		
0	19	31.1
+1	15	24.6
+2	10	16.4
+3	17	27.9
