# Supplementary material for: Enhanced detection of cell-free DNA (cfDNA) enables its use as a reliable biomarker for diagnosis and prognosis of gastric cancer
Source: PLoS One. 2020 Dec 2;15(12):e0242145. doi: 10.1371/journal.pone.0242145 (PMC7710035; doi:10.1371/journal.pone.0242145)
Supplement: S2 Table — (PDF) [file pone.0242145.s005.pdf]

	Pearson's correlation (vs age)	p value
cfDNA, Bead	0.141	0.280
cfDNA, Kit	0.129	0.323
LDH	0.091	0.486
CRP	-0.090	0.491
