# Supplementary material for: Enhanced detection of cell-free DNA (cfDNA) enables its use as a reliable biomarker for diagnosis and prognosis of gastric cancer
Source: PLoS One. 2020 Dec 2;15(12):e0242145. doi: 10.1371/journal.pone.0242145 (PMC7710035; doi:10.1371/journal.pone.0242145)
Supplement: S3 Table — (PDF) [file pone.0242145.s006.pdf]

	Woman (n = 20)	man (n = 41)	p value
cfDNA, Bead (ng/μL)	4.00 ± 6.50	4.19 ± 6.62	0.918
cfDNA, Kit (ng/μL)	2.61 ± 5.81	3.46 ± 6.49	0.624
LDH (U/L)	402 ± 122	385 ± 94	0.553
CRP (ng/μL)	2.56 ± 5.38	2.97 ± 3.63	0.73
