# Supplementary material for: Enhanced detection of cell-free DNA (cfDNA) enables its use as a reliable biomarker for diagnosis and prognosis of gastric cancer
Source: PLoS One. 2020 Dec 2;15(12):e0242145. doi: 10.1371/journal.pone.0242145 (PMC7710035; doi:10.1371/journal.pone.0242145)
Supplement: S4 Table — (PDF) [file pone.0242145.s007.pdf]

	cfDNA, Bead (ng/mL)	cfDNA, Bead (ng/mL)	LDH (U/L)	CRP (ng/µL)	CEA (ng/mL)	CA19-9[#] (ng/mL)
All Patients (n = 61)			387.0 (226.0-742.0)	0.50 (0.10-17.70)	2.58 (0.23-1,500)	8.29 (0.60-117.64)
T Stage						
T2 (n = 12)	78.3 (0 - 275.8)	15.8 (0 - 202.1)	369.5 (267.0-424.0)	0.50 (0.10-7.10)	2.82 (0.46-8.96)	5.63 (1.74-71.13)
T3 (n = 12)	59.6 (0 - 247.7)	155.8 (60.2 - 377.1)	388.0 (226.0-657.0)	0.50 (0.10-17.60)	3.11 (0.87-92.04)	8.03 (5.49-117.64)
T4 (n = 37)	515.8 (98.3 - 12313.3)	130.8 (15.8 - 4590.8)	398.0 (265.0-742.0)	0.50 (0.10-17.70)	2.50 (0.23->1,500)	8.57 (0.60-36.87)
Significance (T2 vs. T3/T2 vs. T4/T3 vs. T4)	.976/ .007/ .018	.072/ .076/ .907	.266/ .250/ .789	.713/ .814/ .543	.719/ .601/ .429	.228/ .731/ .301
N Stage						
N0 (n = 11)	87.1 (17.7 - 273.3)	87.1 (6.5 - 3518.3)	334.0 (267.0-437.0)	0.50 (0.10-8.80)	3.02 (0.46-1500.00)	9.20 (1.74-60.11)
N1 (n = 11)	103.3 (0 - 419.6)	130.8 (15.8 - 234.6)	390.0 (281.0-742.0)	0.50 (0.30-4.70)	2.49 (0.89-8.96)	5.02 (0.60-71.13)
N2 (n = 10)	153.3 (26.5 - 11675.8)	80.8 (5.8 - 8925.8)	408.5 (276.0-657.0)	3.00 (0.10-17.70)	3.29 (0.23-92.04)	14.79 (2.00-36.87)
N3 (n = 29)	515.8 (138.3 - 0)	205.8 (58.3 - 0)	379.0 (226.0-679.0)	0.50 (0.10-17.60)	2.24 (0.66-90.87)	8.29 (0.60-117.64)
Significance (N0 vs. N1/N0 vs. N2/N0 vs. N3/ N1 vs. N2/N1 vs. N3/N2 vs. N3)	.614/ .476/ .040<br>1.000/ .080/ .134	.974/ .618/ .455<br>.620/ .294/ .099	.193/ .123/ .402<br>1.000/ .402/ .437	.438/ .387/ .720<br>.085/ .858/ .196	.797/ .756/ .385<br>.557/ .437/ .174	.468/ .549/ .952<br>.175/ .375/ .480
M Stage						
M0 (n = 50)	180.8 (0 - 515.8)	125.8 (25.8 - 733.3)	388.0 (226.0-742.0)	0.50 (0.10-17.70)	2.60 (0.23-1500.00)	6.97 (0.60-117.64)
M1 (n = 11)	11178.3 (1798.3 - 14580.8)	113.3 (0 - 4839.6)	379.0 (311.0-679.0)	0.50 (0.10-7.90)	2.55 (0.66-90.87)	16.65 (1.74-36.87)
Significance	.008	.644	.896	.777	.750	.221

[#] CA 19-9 was analyzed for 52 patients with gastric tumor.
